# Increasing Whole Grain Intake as Part of Prevention and Treatment of Nonalcoholic Fatty Liver Disease

**DOI:** 10.1155/2013/585876

**Published:** 2013-05-16

**Authors:** Alastair B. Ross, Jean-Philippe Godin, Kaori Minehira, John P. Kirwan

**Affiliations:** ^1^Nestlé Research Center, Vers chez les Blanc, 1000 Lausanne 26, Switzerland; ^2^Chalmers University of Technology, 412 96 Gothenburg, Sweden; ^3^Lerner Research Institute, Cleveland Clinic, 9500 Euclid Avenue Cleveland, OH 44195, USA

## Abstract

In conjunction with the rise in rates of obesity, there has been an increase in the rate of nonalcoholic fatty liver disease (NAFLD). While NAFLD at least partially originates from poor diet, there is a lack of nutritional recommendations for patients with suspected or confirmed diagnosis of NAFLD, beyond eating a healthy diet, increasing physical activity, and emphasising weight loss. The limited current literature suggests that there may be opportunities to provide more tailored dietary advice for people diagnosed with or at risk of NAFLD. Epidemiological studies consistently find associations between whole grain intake and a reduced risk of obesity and related diseases, yet no work has been done on the potential of whole grains to prevent and/or be a part of the treatment for fatty liver diseases. In this review, we examine the potential and the current evidence for whole grains having an impact on NAFLD. Due to their nutrient and phytochemical composition, switching from consuming mainly refined grains to whole grains should be considered as part of the nutritional guidelines for patients diagnosed with or at risk for fatty liver disease.

## 1. Introduction

Nonalcoholic fatty liver disease (NAFLD) is one of the major liver diseases worldwide. Estimates of average prevalence vary from around 20–46% of the adult population in Westernised countries [1–3], with up to 45% in some ethnic groups [3, 4]. The prevalence of NAFLD increases up to 90% in obese populations, and given the worldwide rise in obesity, the incidence of NAFLD is likely to grow over the coming decades. While hepatic steatosis, an early stage of NAFLD, is often asymptomatic, in 20–30% of all cases, it can progress to nonalcoholic steatohepatitis (NASH). If untreated, NASH can progress to hepatic cirrhosis and reduced liver function and increased risk of early mortality [5]. Hepatic steatosis is characterised by a large number of fatty deposits in the liver. Critically, NAFLD in the hepatic steatosis phase can be reversed by lifestyle modification, while NASH is more difficult to treat though it can be reversed by bariatric surgery [6]. Thus, preventing the progression of hepatic steatosis to NASH is of primary importance.

Insulin resistance and oxidative stress are currently considered the primary mediators of NASH [7]. Other causes of NASH include total parenteral nutrition, certain drugs and industrial toxins, copper toxicity, and conditions characterized by extreme insulin resistance [8]. Peripheral insulin resistance leads to increased delivery of fatty acids to the liver, and high levels of circulating insulin interfere with the normal capacity of hepatic mitochondrial *β*-oxidation to metabolize fatty acids; these steps in turn lead to fat accumulation in the liver [9]. NASH most likely develops from a subsequent “second hit” whereby oxidative stress leads to lipid peroxidation in the liver [10]. Mitochondrial dysfunction and, more specifically, respiratory chain deficiency may also mediate the second hit by generating reactive oxygen species which oxidise fat deposits to release lipid peroxidation products toxic to hepatocytes and other hepatic cells [11]. The increase in oxidative stress and lipid peroxidation leads to greater inflammation, leading to activation of tumour necrosis factor-*α* and interleukin-6 mediated pathways and down regulation of hepatocyte autophagy (mitophagy, i.e., including the process of removing damaged mitochondria) [12].

While hepatic steatosis in itself may not lead to noticeable symptoms, the development of NASH is associated with significant morbidity and mortality and has limited therapeutic options [13–15]. The main causes of increased mortality among patients with NASH are cardiovascular disease, cancer, and liver failure [12]. Patients with NAFLD are at increased risk for other chronic diseases, including type 2 diabetes and cardiovascular disease [16, 17].

Key risk factors for NAFLD include (1) positive energy balance (excess energy intake and/or reduced physical activity), (2) obesity, (3) insulin resistance, and (4) hypertriglyceridemia. These factors are key targets for prevention and therapy of NAFLD though clinical treatment tends to focus on addressing the obesity or insulin resistance rather than NAFLD itself. As with all lifestyle-related diseases, improvement of diet should also play a role in its prevention and treatment.

The clinical diagnosis of NAFLD requires a liver biopsy; however, blood biomarkers (alanine aminotransferase and aspartate aminotransferase) [25] are commonly used in large-scale epidemiological studies that have examined the link between diet composition and NAFLD. While these measures have low specificity, they provide some indirect insight into the effects of diet on liver function. Presently, it is known that a high percentage of energy from fat, and especially saturated fat, may lead to liver damage and that high glycaemic foods and refined sugar and fructose may also lead to hepatic steatosis through increased de novo lipogenesis [26]. There is mixed evidence on the role of vitamins, with some suggestions that better vitamin D and E status is linked to lower incidence of NAFLD, but intervention studies that have examined the effects of vitamin E supplementation on NASH did not show conclusive improvement [7, 27, 28]. These studies have been supplemented with *α*-tocopherol in an attempt to treat NASH, which does not rule out a role for other E vitamers and prevention or treatment of NAFLD. Studies that have investigated the effects of dietary fat and carbohydrate have shown that isoenergetic diets with low-fat and low-carbohydrate diets decrease liver fat, while high-fat diets increase liver fat over two weeks [29, 30], and increased protein intake may also reduce intrahepatic lipids [31]. The mechanisms behind these effects are not well understood, and the long-term efficacy/safety of low-carbohydrate and high-protein diets has not been assessed although present clinical recommendations state that ketogenic diets should be avoided to prevent greater liver damage. A Mediterranean diet rich in vegetables, nonred meat protein sources, and polyunsaturated fat was found to improve insulin sensitivity and reduce liver fat without weight loss over 6 weeks [32], and a short-term exercise intervention reduced markers of NAFLD and apoptosis (alanine aminotransferase and cytokeratin 18), also without significant weight loss [33], suggesting that it may be possible to improve NAFLD without losing weight. Omega-3 fatty acid supplementation has also been used as a treatment for NAFLD, with some promising results [26], but it is outside the scope of this review.

Despite the strong recent efforts in the area of diet and NAFLD, present lifestyle recommendations for people diagnosed with NAFLD are generally limited to losing weight (e.g., through energy restriction) and increasing physical activity [34], as well as following national guidelines for a healthy diet [2, 35]. In many countries, this now includes the recommendation to eat at least half of all grain servings as whole grains, with gram recommendations ranging from at least 48 g/d to at least 75 g/2000 kcal [36]. 

While there are reviews that have covered the area of diet and physical activity for the treatment of NAFLD [26, 37, 38], none have examined whether increasing whole grains in the diet may be an effective strategy for prevention and treatment of NAFLD. This review aims to examine the evidence supporting the idea that a diet rich in whole grains is associated with a decrease in many of the risk factors and comorbidities associated with NAFLD and that replacing refined grains with whole grains in the diet could also be an important component of lifestyle changes that could successfully prevent and treat NAFLD.

## 2. Whole Grains

Whole grain cereals (whole grains; WG) are consistently associated with a decreased risk of NAFLD-related diseases, including obesity, diabetes, and cardiovascular disease [39]. The term “whole grains” covers the edible parts of the cereal grasses, including wheat, rice, corn, rye, barley, and oats, and also includes the pseudocereals (seeds that are used in a similar manner to cereal grains) (Table 1). The American Association of Cereal Chemists defines whole grains as cereals that “… shall consist of the intact, ground, cracked, or flaked caryopsis, whose principal anatomical components—the starchy endosperm, germ, and bran—are present in the same relative proportions as they exist in the intact caryopsis” [40]. Over the past 100–150 years, the most commonly consumed cereals are milled to remove the bran and the germ from the starch endosperm. This results in flour that has a longer shelf-life, has better organoleptic properties, and is easier to process than if all three components are present [41]. This also results in flour which is nutrient poor relative to the whole grain, especially in dietary fibre, vitamins, and minerals, as well as phytochemicals that may have health benefits (Table 1). Cereal grains represent one of the main staple foods worldwide, and improving the quality of cereal foods in the diet represents an excellent opportunity for improving health. 

While several national dietary guidelines now include recommendations for increasing whole grain intake and replacing refined grains with whole grains [36], the average consumption remains relatively low at around one 16 g serving/d [42–44]. It should be noted that these data are from surveys taken before the 2005 Dietary Guidelines for Americans, which placed an increased emphasis on whole grains [39]. Thus, even though there is an increasing availability of a wide variety of whole grain foods and greater public awareness of the potential health benefits of whole grains in the diet, recent data suggests that this has not been translated into greater whole grain consumption [45].

## 3. Whole Grains and Risk Factors for NAFLD 

A considerable body of epidemiological work outlined below consistently reports that people who eat more whole grains have a reduced risk of cardiovascular disease, obesity, and diabetes, while some studies suggest that whole grains may also reduce markers of inflammation.

Several meta-analyses of epidemiological data have found that increasing whole grain intake reduces the risk of developing type 2 diabetes in the order of 16–26% compared to people eating the least amount of whole grains in their diet [20, 22, 46] (Table 2). Greater intake of whole grains is also associated with lower fasting C-peptide and insulin although not glycated haemoglobin (HbA1c) [47]. Recently, greater whole grain intake was associated with a 34% lower risk of deteriorating glucose tolerance [48].

A high whole grain intake is also linked to a decreased likelihood of being obese [49, 50]. There is evidence from both epidemiological [51] and randomised intervention trials [52, 53] that abdominal fat mass is reduced on a whole grain diet compared to refined grains, an effect which has not been reported for fruits and vegetables [51]. In a meta-analysis of three major US cohorts, a single serving (assumed to be 16 g) with an increase in whole grain intake resulted in a 0.25 kg decrease in body weight over four years, a small but nevertheless significant change [24]. Eating whole grains instead of refined grains while on a hypoenergetic diet does not increase weight loss although it does appear to improve fat loss [52, 54]. For NAFLD, reduction of body fat, rather than overall weight loss, may be more important, making these findings supporting the use of whole grains as a carbohydrate source during hypoenergetic diets highly interesting. Mechanisms behind whole grains leading to preferential loss of body fat remain to be determined.

There are some reports that whole grain intake correlates with reduced concentrations of some inflammation markers [55, 56] although this is not a universal finding [47]. Consuming >1 serving of whole grain was associated with reduced high-sensitivity C-reactive protein (hsCRP) in premenopausal women [56], while plasminogen activator inhibitor type 1 (PAI 1) and hsCRP were also decreased in adults who ate the most whole grains though fibrinogen was not associated with whole grain intake [55]. In women, whole grain intake was associated with reduced plasma CRP and tumour necrosis factor *α*-receptor 2; these effects were attenuated by waist circumference, insulin sensitivity, and 2 h-postload glucose [57], suggesting a strong link between inflammation and body composition/insulin resistance. Overall, epidemiological studies suggest that each 16 g serving of whole grain leads to a 7% decrease in CRP [58]. Evidence from intervention trials is less convincing, with one study finding reduced CRP in subjects with metabolic syndrome on a hypocaloric diet while eating whole grains compared to refined grains [52], and another finding IL-6 concentrations decreased, especially in overweight and female subjects as they had higher baseline IL-6 concentrations [59]. However, most other studies that have examined inflammation parameters have not found significant differences between refined grain versus whole grain consumption [60–62]. 

While epidemiological studies overwhelmingly report associations between highest intake of whole grains and reduced risk of cardiovascular diseases, diabetes, and obesity compared to those eating little or no whole grains [20, 21, 39], it should be acknowledged that intervention evidence for the effect of whole grains on markers of disease risk is mixed, with a number of studies finding a range of positive biomarker changes when eating whole grains compared to refined grains [52–54, 59, 62–66] while other studies have not found any benefits [60, 61, 67]. In some cases, this may be due to the difficulty of ensuring compliance in free-living settings [68], as well as the considerable heterogeneity of study design and populations used [20]. Meta-analyses of epidemiological and clinical trials point to an overall effect of diets rich in whole grains rather than refined grains reducing many of the risk factors for NAFLD (Table 2).

## 4. Potential Mechanisms of Action for Whole Grains in the Prevention and Treatment of NAFLD

Whole grains are higher in many nutrients and phytochemicals than their refined counterparts (Table 1), and several reviews have described possible mechanisms behind how they may be better for health [69, 70]. In the context of prevention or treatment of NAFLD, there are several possible mechanisms beyond better nutrient intake:

- reduction of energy intake (lower energy density compared to refined foods),
- changes to and stimulation of gut microbiota, leading to increased production of short-chain fatty acids,
- specific actions of phytochemicals (e.g., vitamins, phenolic acids, betaine),
- synergistic interaction between different whole grain components (e.g., phenolic compounds interacting with stimulated gut microbiota).

In general, whole grain foods are less energy dense than their refined counterparts, though this depends on the amount of lipid from the germ; refined grains have the germ removed and a lower overall fat content. However, lipids from cereals are mainly unsaturated and so are not a primary cause for concern for the development of NAFLD. The slightly lower (around 5%) amount of carbohydrate in whole grains compared to refined grains may also reduce the amount of insulin required to handle the influx of glucose after a meal. The higher amount of fibre has also been suggested to increase intestinal bulking and thus influence satiety. There is little data to support an impact of whole grains on satiety [71], with the exception of rye [72–75]. However, whole grains are perceived as being more satiating by consumers [76], and this over the long term may reduce energy intake by reducing portion sizes.

Several studies have now demonstrated that whole grains can change gut microbiota composition in humans, including *Bifidobacteria* and *Lactobacillus* [59, 66, 77, 78], with corresponding changes to gut microbiota metabolites including the short-chain fatty acid (SCFA) butyrate measured in faeces [79, 80] and plasma [81] and phenolic compounds measured in urine [79, 80]. Increased butyrate production is linked to improved tissue insulin sensitivity [82–84], and this may be one mechanism for how whole grains improve insulin sensitivity (see below). Increasing numbers of studies have found links between gut microbiota composition/metabolism and fatty liver disease [85–88], particularly for the role of lipopolysaccharide (LPS; a cell wall component of gram negative bacteria) and increased inflammation [89] and risk for NAFLD and NASH [90, 91]. Prevention of LPS uptake from the intestine may be limited by increased intestinal permeability; subjects with NAFLD or NASH are more likely to have increased intestinal permeability compared to healthy controls [92, 93]. LPS-induced inflammation via toll-like receptors has been linked to obesity and is proposed as a factor in the “second hit” of NAFLD leading to NASH [12]. Low-dose exposure to LPS may also increase hepatic triglyceride production and inhibit triglyceride export at higher doses [94] through inflammation-mediated interruption of fatty acid transporters [95]. Recently, it was proposed that increased circulating leptin leads to a hypersensitive response to circulating LPS [96], underlining the multitude of factors influencing the response to gut microbiota components. In a rat model of alcoholic fatty liver disease, rats fed with an oat-based diet had less liver fat accumulation than controls, and this effect was ascribed to thickening of the intestinal wall, thus preventing excessive ethanol transport across the intestine [97]. This mechanism may also inhibit transport of LPS across the intestinal barrier and prevent endotoxin-associated inflammation. Prebiotic fibre has been linked to changes in gut microbiota and NAFLD, and this may serve as another potential mechanism whereby whole grains protect against NAFLD. The addition of fructooligosaccharide (FOS) to the diet of mice with fatty liver induced by n-3 polyunsaturated fatty acid deficiency resulted in altered gut microbiota populations (including increased *Bifidobacteria*, as for some whole grain clinical studies [59, 77]) and reduced hepatic triglyceride accumulation. This effect was mediated via proliferator-activated receptor *α*-stimulation of fatty acid oxidation, possibly stimulated by the gut hormone GLP-1 [98]. The composition of gut microbiota and the integrity of the intestinal barrier may be key factors in the interaction between diet and NAFLD [89]. Diets rich in whole grains appear to consistently alter gut microbiota composition. Beyond the study of Keshavarzian et al. [97], no studies have examined whether whole grains can improve gut integrity. 

A number of studies have demonstrated lower glycemic curves following a whole grain diet relative to refined wheat [81, 99], and the rate of glucose disappearance was increased in a morning glucose tolerance test after an evening meal of whole grain barley compared to white rice [100], possibly indicating improved insulin sensitivity. Beta-cell function was also found to be improved after a whole grain rye-based intervention [101]. These apparent improvements in glucose metabolism may be due to increased fermentation activity of the gut microbiota, through the action of butyrate, or due to micronutrients such as magnesium that are present in relatively high amounts in whole grain. The latter serves as cofactors for enzymes involved in glucose metabolism and insulin secretion [102]. It should also be noted that not all intervention studies have found improvements in markers of glucose metabolism (e.g., [60, 61]), and the outcomes may be highly dependent on the study population and design. 

There are many phytochemicals that are abundant in whole grains but are only present in low amounts after refining. Of particular interest for potential treatment of NAFLD, whole grain wheat, rye, and quinoa have high amounts of the methyl donor glycine betaine (trimethylglycine, referred to henceforth by its common name “betaine”) compared to other foods [19, 103]. High doses of oral betaine have been shown to reduce the severity of NAFLD in humans in case studies [104–106]. However, in one randomised controlled trial testing betaine supplementation there was no conclusive evidence for an effect on NASH or inflammation although betaine did reduce the steatosis grade [107]. The rationale for the effect of betaine is that it spares choline (which can be metabolised to betaine for remethylation of homocysteine) for synthesis into phosphatidylcholine, which in turn is a key component of VLDL particles for export of lipids from the liver. If too much choline is required for remethylation of homocysteine, then export of VLDL from the liver will be compromised, leading to a buildup of lipids in the liver. This type of mechanism has been demonstrated in rodent models of NAFLD where betaine supplementation reduces the severity of fatty liver in NAFLD models [108]. Higher plasma homocysteine itself has also been associated with steatosis and NASH [109, 110], and rodent studies suggest that Hyperhomocysteinaemia may cause liver damage and peroxidation [111]. However, elevated plasma homocysteine has not been associated with steatosis or NASH in all populations [112, 113], and genetics may play a role in determining whether hypohomocysteinaemia is a risk factor, especially for the progression to liver cirrhosis [114]. Whether betaine supplementation is effective in reducing steatosis or NASH in cases of hyperhomocysteinaemia is yet to be tested.

**Figure 1:**
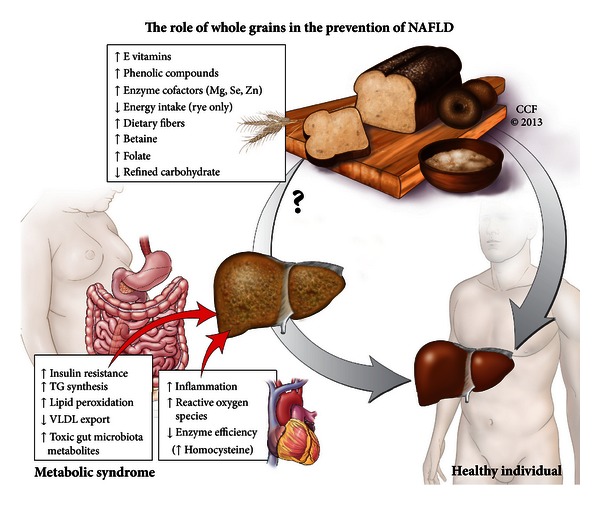
Whole grains may have an impact on nonalcoholic fatty liver disease through many complementary mechanisms. Choosing more whole grains over refined carbohydrate sources will increase the intake of many nutrients that are known to, or suggested to, play a role in preventing fatty liver diseases and related comorbidities. While yet to be studied directly, it is probable that a diet rich in whole grains would play a role in the prevention of fatty liver diseases. Whether they could be a biologically active part of a diet to treat nonalcoholic fatty liver disease remains to be investigated.

Wheat-based foods are the main source of betaine in the diet and account for 67% of total betaine intake in the New Zealand diet [103]. Choosing whole grains over refined grains could increase overall betaine intake by 1.5–3.3 fold. Although there are no existing recommendations for betaine intake, choline intake recommendations for adults in the US are 425–550 mg/d [115], recognizing that the pathway between phospholipid synthesis and remethylation of homocysteine is of importance for general health. It is uncertain what amount of dietary betaine is required to maintain optimal betaine status and this is likely to depend on choline and B-vitamin status as well. A whole grain rich diet (150 g/d) with 112 mg/d more betaine than the control refined grain diet increased plasma betaine [66] and a very high amount of wheat aleurone fraction (providing 279 mg/d more betaine than the control) reduced homocysteine and increased betaine, dimethylglycine, and methionine in healthy adults [116]. In epidemiology, greater whole grain intake has also been associated with decreased homocysteine [47]. Low plasma betaine and high plasma choline were associated with increased BMI, body fat, and serum triglyceride, while increased plasma betaine was associated with increased plasma folate, serum HDL, and decreased plasma homocysteine [117]. The one-carbon metabolic pathway may also interact with the gut microbiota, which may also play an important role in the development of fatty liver in humans on a choline deficient diet [118]. Given that betaine reverses fatty liver in rodents on a choline-deficient diet, whole grains as a main source of dietary betaine and an ingredient that can alter gut microbiota, mean that several different but interlinked mechanisms for preventing fatty liver may potentially occur (Figure 1). Independent of whole grain intake, coeliac disease may be a risk factor for NAFLD, and elevated concentrations of transaminases are common in people with coeliac disease [119] though this could also be due to intestinal damage. The gluten-induced gastrointestinal inflammation is hypothesised to lead to increased gut leakage [91] and increased blood LPS, and people diagnosed with NAFLD and coeliac disease often improve after they are placed on a gluten-free diet. Monitoring coeliac disease is suggested for NAFLD patients, even when no other metabolic risk factor is present [119]. This being the case, it should be noted that cereals that form the basis of gluten-free diets (i.e., avoidance of wheat, rye, barley and oats) are generally very low in betaine ([19] and Table 1), with the exception of the South American pseudocereals amaranth and quinoa. In the case of individuals with coeliac disease or gluten intolerance who are also at risk of NAFLD, regular consumption of these two pseudocereals may be advisable. 

Recently, Fardet proposed the concept of lipotropes, compounds that may play a role in preventing excessive hepatic fat accumulation [120], including betaine, choline, myo-inositol, methionine, magnesium, niacin, pantothenic acid, folate, and total polyphenol content. Cereal-based foods, and especially whole grains, are considered the most economic source of lipotropic compounds [121]. However, this hypothesis is currently based on animal data and it remains to be seen if the lipotropic index translates it to the clinical setting. Nevertheless it is clear that whole grains are always higher in these “lipotropic” compounds compared to their refined equivalents, and when cereals make up a large part of our energy intake, this may have consequences for NAFLD. 

Other compounds present in high amounts in cereals may play a role in preventing NAFLD. In particular, whole grains are rich in phenolic compounds, mainly ferulic acid. *In vitro* and animal studies suggest that ferulic acid conjugates can reduce inflammation via inhibition of the transcription factor NF-*κ*B and activation of proinflammatory pathways [122]. However, ferulic acid itself had little effect in a Caco-2 cell model of intestinal inflammation though flavonoids did [123]. A fermented whole grain wheat bread was found to reduce *ex-vivo* cytokine production in blood, and this was associated with greater ferulic acid bioavailability [124]. In cereals, the phenolic acids are mainly (>95%) bound to fibre and are not thought to be released from the matrix during intestinal transit, thus leading to a low bioavailability of <5% [125]. It is possible that even if phenolic acids are not absorbed, they do play a role in modulating gut microbiota and improving the chemistry of the intestinal milieu [126]. While ferulic acid is a strong antioxidant, it is unlikely that the amount absorbed has a meaningful effect on overall antioxidant status through classical mechanisms [127, 128]. Another group of phenolic compounds, the alkylresorcinols (phenolic lipids), is present in high amounts in wheat and rye and has around 60% absorption by humans [129]. There is relatively little information about any bioactivity *in vivo* [130] although one study where rats were fed rye alkylresorcinols found that high doses (0.4% of total diet) led to a dramatic decrease in total liver lipids and an increase in liver *γ*-tocopherol via competitive inhibition of CYP450-mediated *β*-oxidation [131]. In adipocyte models, alkylresorcinols were also found to inhibit triglyceride synthesis [132] and inhibit hormone sensitive lipase-mediated-lipolysis of triglycerides [133], suggesting that they may both prevent excessive triglyceride accumulation and prevent elevated circulating concentrations of nonesterified fatty acids (NEFA) [134]. Alkylresorcinol intake was associated with decreased plasma NEFA after a whole grain intervention [68] though more *in vitro* and *in vivo* studies are needed to confirm this potential mechanism. Oats contain phenolic analogues of the anti-inflammatory drug tranilast, avenanthramides. Avenanthramides also have anti-inflammatory properties *in vivo* [135], though concentrations in oats are low, and a high intake of oats would likely be required to observe an acute effect. Avenanthramides may in part be responsible for the effect of oats in protecting against ethanol damage to the liver of rodents, otherwise ascribed to an improvement of the intestinal barrier [97].

It is also likely that the different components of whole grains act together synergistically. A whole grain wheat diet increased faecal Bifidobacteria populations in humans compared to wheat bran [77], and whole grain wheat decreased cholesterol in a rat model, whereas wheat bran did not [136]. It is also speculated that phenolic compounds and dietary fibre from whole grains differentially interact with the host microbiota to improve overall host-microbiota interactions [126]. The synergy between the different components of the whole grain may account for why they are associated with reduced risk of a wide range of diseases.

Whole grain foods potentially contain a variety of anti-nutrients and toxins, as the outer protective layer of the grain is included, and this is exposed to soil, fungal infections, and pesticides. In practice, cereal raw materials are strictly monitored to ensure that known mycotoxins and pesticides are below local thresholds of concern [137, 138], and millers often remove the outer 1-2% of the grain before making “whole grain” flour to substantially reduce the likelihood of contaminants being present [139]. Whole grains are also high in phytate, which decreases mineral bioavailability, both micronutrient minerals and heavy metals [140], and rice bran phytate has been demonstrated to reduce lead poisoning in a rat model [141]. While contaminated grains could be a source of toxins that could cause liver damage, there is no population-based evidence to suggest that current thresholds for monitoring contaminants are inadequate.

In the diet and NAFLD literature, there is some concern that diets higher in carbohydrates may be linked to the increased incidence of NAFLD [142, 143] and that low-carbohydrate diets may lead to greater fat loss and improvement in insulin sensitivity [29, 144]. However, these studies do not describe the type of carbohydrate in the diet, nor the origins. While sucrose, refined white wheat flour, whole grain flour, and potatoes may all be classed as “carbohydrate rich”, they all lead to different metabolic responses, even before accounting for different food processing methods. We therefore suggest that the source of carbohydrates may be critical and that the greater nutrient content of whole grains makes them a “positive” source of energy for people with NAFLD compared to other carbohydrate rich foods that are dietary staples in the Western diet and may exacerbate fatty liver disease.

## 5. Conclusions

Presently the only empirically based dietary advice for patients with NAFLD is to eat a hypoenergetic diet and lose weight, and there is little evidence for what sort of foods should be used to achieve this with the best outcome. People who consume diets rich in whole grains tend to have a lower risk of many of the comorbidities associated with NAFLD, including NASH, and all whole grains contain higher amounts of compounds which may help reduce liver fat and protect against the inflammation that is thought to act as the second hit that leads to the progression from steatosis to NASH. Since there is no direct evidence that a whole grain diet may help to protect or treat NAFLD, clinical studies comparing whole grains with refined grains for preventing and treating NAFLD are needed. Given the multiple potential mechanisms where whole grains may prevent or treat risk factors associated with NAFLD (Figure 1), there is a good basis to recommend that people with or at risk of NAFLD should choose whole grains over refined grains in their diet.

## Figures and Tables

**Table 1:** Whole grains included under the American Association of Cereal Chemists definition, key macronutrients, and micronutrients that may play a role in nonalcoholic fatty liver disease. Refined wheat and rice are included as comparisons. Data are from the USDA database [18] unless otherwise stated. Note that these values are averages and do not represent the high varietal and seasonal variation that are normal for micronutrient contents of foods.

	Whole grains											Refined grains		
	Wheat	Rice	Corn	Rye	Oats	Barley	Sorghum	Millet	Quinoa^a^	Buckwheat^a^	Amaranth^a^	Wheat	Rice	Corn
Energy (kJ/100 g)	1418	1515	1515	1414	1628	1481	1418	1582	1540	1402	1552	1523	1498	1569
Carbohydrate (g/100 g)	72.6	76.2	76.9	75.9	66.3	73.5	74.6	72.9	64.2	70.6	65.3	76.3	79.2	82.8
Protein (g/100 g)	13.7	7.5	8.1	10.3	16.9	12.5	11.3	11	14.1	12.6	13.6	10.3	6.5	5.6
Fat (g/100 g)	1.9	2.7	3.6	1.6	6.9	2.3	3.3	4.2	6.1	3.1	7	1	0.5	1.4
Total dietary fibre (g/100 g)	12.2	3.4	7.3	15.1	10.6	17.3	6.3	8.5	7	10	6.7	3.1	1	1.9
Vitamin E (mg *α*-tocopherol/100 g)	0.8	0.6	0.4	0.9	0.7	0.6	0.1	0.1	2.4	0.3	1.2	0.1	0.1	0.2
Folate (*µ*g/100 g)	44	20	25	38	56	19	—	85	184	54	82	10	6	48
Magnesium (mg/100 g)	138	143	127	110	177	133	—	114	197	251	248	22	35	18
Glycine betaine (mg/100 g)^b^	90	3	2	120	7	35	3	10	360	2	65	23	3	3
Free choline (mg/100 g)^b^	20	8	2	18	4	7	10	2	27	46	51	10	10	18

^a^Pseudocereals: botanically not true cereal grasses, but included in the whole grain definition due to their traditional use in the same way as cereals.

^b^Data from Bruce et al. [19] and unpublished results using the same liquid chromatography-tandem mass spectrometry method.

**Table 2:** Associations between whole grain intake with risk factors for nonalcoholic fatty liver disease. Data are from meta-analyses only.

	Relative risk ratio/weighted mean difference compared to controls*	*P* value (after adjustment for potential confounders unless otherwise stated)	Median or average whole grain intake (high versus low; g whole grain/d)	Whole grains consumed	Study type	Number of cohorts/studies included in the meta-analysis	Reference
Cardiovascular disease (incidence)	0.79 (0.74, 0.85)	<0.001	44 versus 0	Mixed (mainly US studies)	Prospective cohort	9	[20]
	0.79 (0.73, 0.85)	<0.001	40 versus 3.2			7	[21]
Type 2 diabetes (incidence)	0.74 (0.69, 0.80)	<0.001	44 versus 0	Mixed (mainly US studies)	Prospective cohort	6	[20]
	0.79 (0.72, 0.87)	<0.001	32 versus 0+	Mixed (mainly US studies)	Prospective cohort	6	[22]
Fasting insulin (pmol/L)	−0.29 (−0.59, 0.01)	<0.001	>50 versus <20	Mixed	Intervention	10	[20]
	−0.011 (−0.015, −0.007)	<0.001	16 versus 0+	Mixed	Prospective cohort	14	[23]
Fasting glucose (mmol/L)	−0.93 (−1.65, −0.21)	<0.001	>50 versus <20	Mixed	Intervention	11	[20]
	−0.009 (−0.013, −0.005)	<0.001	16 versus 0+	Mixed	Prospective cohort	14	[23]
Total cholesterol (mmol/L)	−0.83 (−1.24, −0.42)	<0.001	>50 versus <20	Mixed	Intervention	16	[20]
LDL-cholesterol (mmol/L)	−0.72 (−1.34, −0.11)	<0.001	>50 versus <20	Mixed	Intervention	15	[20]
Weight gain (kg)	−0.18 (−0.54, 0.18)	ns	>50 versus <20	Mixed	Intervention	12	[20]
	−0.17 (−0.22, −0.11)	<0.001	16 versus 0+	Mixed (US cohorts)	Prospective cohort	3	[24]

*Highest versus lowest categories of whole grain intake in prospective cohort studies and weighted mean difference compared to controls in intervention studies.

**These values are not actual intake but are the difference intake estimated to lead to the corresponding change in biomarker concentration.
